# Combating bone marrow failure with polymer materials

**DOI:** 10.3389/fimmu.2024.1396486

**Published:** 2024-04-17

**Authors:** Kayla C. Koch, Nidhi Jadon, Iris Thesmar, Gregory N. Tew, Lisa M. Minter

**Affiliations:** ^1^ Department of Polymer Science and Engineering, University of Massachusetts Amherst, Amherst, MA, United States; ^2^ Department of Veterinary and Animal Sciences, University of Massachusetts Amherst, Amherst, MA, United States; ^3^ University of Massachusetts Amherst, Amherst, MA, United States

**Keywords:** cell expansion, polymer hydrogel, drug delivery, gene delivery, cell penetrating peptides, bone marrow failure

## Abstract

Bone marrow failure (BMF) has become one of the most studied autoimmune disorders, particularly due to its prevalence both as an inherited disease, but also as a result of chemotherapies. BMF is associated with severe symptoms such as bleeding episodes and susceptibility to infections, and often has underlying characteristics, such as anemia, thrombocytopenia, and neutropenia. The current treatment landscape for BMF requires stem cell transplantation or chemotherapies to induce immune suppression. However, there is limited donor cell availability or dose related toxicity associated with these treatments. Optimizing these treatments has become a necessity. Polymer-based materials have become increasingly popular, as current research efforts are focused on synthesizing novel cell matrices for stem cell expansion to solve limited donor cell availability, as well as applying polymer delivery vehicles to intracellularly deliver cargo that can aid in immunosuppression. Here, we discuss the importance and impact of polymer materials to enhance therapeutics in the context of BMF.

## 1 Introduction

Bone marrow failure (BMF) is characterized by the immune-mediated destruction of hematopoietic stem cells (HSCs) in the bone marrow. This results in a reduced number of hematopoietic precursors and subsequent cytopenias. BMF can be classified into two types: acquired and inherited. Inherited bone marrow failure (IBMF) refers to the failure of bone marrow that is caused by genetic mutations inherited from parents or arising spontaneously. Alongside the common symptoms of aplastic anemia, such as fatigue, bleeding episodes, and recurring bacterial infections, patients with IBMF often exhibit additional features specific to each syndrome (1). On the other hand, acquired bone marrow failure is primarily idiopathic in nature. The first line of treatment is to undergo a hematopoietic stem cell transplantation (HSCT) from a matched sibling donor. The first alternative for patients without a matching sibling donor is a matched unrelated donor (MUD) at the allele level (2). Survival rates after matched sibling or MUD hematopoietic stem cell transplantation (HSCT) for aplastic anemia (AA) are currently reported to be around 80% or even higher. Since AA is a nonmalignant hematologic disorder, the risk of relapse after HSCT is generally low. However, graft-versus-host disease (GVHD) remains a significant cause of morbidity and mortality in AA patients who undergo HSCT. GVHD is a complication after HSCT when the graft’s immune cells recognize the host as foreign and attack the recipient’s cells and tissues. GVHD can affect various target organs, such as the skin and lungs, contributing to long-term complications and further posing risks to the overall health of the patients (3–5). Approximately 60-70% of patients do not find a matching unrelated donor. Therefore, there is an ongoing need for alternative HSCTs options (6, 7). One possibility is to utilize a human leukocyte antigen (HLA)-mismatched unrelated donor whose genetic makeup differs from the patient at one allele. Another option involves utilizing umbilical cord-blood (CB) from unrelated donors, or haploidentical (haplo) familial donors which offers greater flexibility in terms of HLA compatibility (8). Alternative HSCTs can potentially provide a curative solution for certain patients. However, it is important to consider that these alternative methods carry higher risks compared to matched sibling or matched unrelated donor HSCTs. These risks include graft rejection, infectious complications, and GVHD. Factors such as patient age, comorbidities, and specificities related to the alternative HSCT methods are important issues in transplantation decision (9).

Recent advancements in cord-blood transplantation have broadened its potential applications by incorporating techniques such as double cord-blood grafts and ex vivo expansion methods (10). In a study performed by Milano et al. (2016), patients with pre-transplantation minimal residual disease had a higher likelihood of overall survival when they received a transplant from a cord-blood donor compared to receiving a transplant from an HLA-matched unrelated donor. Additionally, the cord-blood group had a lower probability of relapse compared to graft recipients from HLA-matched unrelated donors or HLA-mismatched unrelated donors (11). Although cord blood is a viable option, its availability remains scarce.

Other therapies for BMF also include drugs that can stimulate colony factors, such as sargramostim (Leukine), filgrastim (Neupogen), pegfilgrastim (Neulasta), and epoetin alfa (Epogen/Procrit), all of which are approved by the United States Food and Drug Administration (USFDA). Additionally, newer treatments such as Eltrombopag increase the quantity of red blood cells and enhance hematopoietic stem cells recovery. Apart from utilizing drugs to improve anemia and aid in BM repopulation, it is also crucial to suppress autoreactive immune cells. Immunosuppressive therapies (IST) typically eliminate auto-reactive T lymphocytes, which are the most common cells to attack HSCs. The USFDA has approved two main IST drugs: anti-thymocyte globulin (ATG) and cyclosporine (12, 13). Although immunosuppressive drugs can enhance patients’ life expectancy, many eventually develop resistance to this treatment and experience dose limitations due to toxicity. Despite patients mainly suffering from liver toxicity and kidney failure, infections and pneumonia associated with IST are the leading cause of deaths among BMF patients (12).

HSCT from a related or unrelated donor can lead to high survival rates, it is important to note that these results are dependent on age. Survival outcomes vary based on age, with older patients >40 years experiencing the least favorable results with HSCTs or ISTs. Additionally, there is a reported risk of older patients >34 years developing GVHD. While IST therapy can provide sustained remission, it is associated with a risk of relapse and late clonal abnormalities. The decision should also consider whether the patient has other health issues (comorbidities). If HSCT is likely to interfere with these other health issues, then ISTs would be the preferred line of treatment as they offer sustained remission (9).

In the absence of next generation drug and transplant therapies, polymeric materials have the potential to revolutionize the BMF therapeutic landscape. Polymers can be employed as novel cell culture matrices to successfully expand hematopoietic stem and progenitor cells (HSPCs) (14, 15). Additionally, there are polymer-based delivery vehicles that can efficiently intracellularly deliver diverse cargo, including antibodies to interrupt signaling pathways (16) or gene editing components to impede target gene transcription in pathogenic T cells (17). These strategies present a promising alternative avenue for traditional HSCT and immunosuppression therapies.

## 2 Polymer materials to enhance current therapeutics

Polymer based materials have been used in diverse biological applications, ranging from therapeutic delivery agents (18), sensors (19), implants (20), and imaging tools (21). Their popularity is mainly attributed to their easily tunable chemistry, architecture, and relatively simple synthesis techniques (22–24). Recent work has shown that three dimensional (3D) polymer matrices are better for cell culturing and expansion than their two dimensional counterparts (i.e. polystyrene plates) (25). Polymer materials are also attractive for intracellular drug delivery applications as they are capable of delivering diverse cargo (antibodies, proteins, genetic material, small molecule therapeutics, etc.) while also generally improving cargo performance (pharmacokinetics) (26–28). Polymer materials have the potential to significantly improve the BMF treatment landscape by facilitating stem cell expansion and intracellular therapeutic agent delivery.

### 2.1 Stem cell expansion

Hematopoietic stem cell destruction is the hallmark of BMF (1). Symptoms of BMF, such as anemia and infections, can be reduced if the BM niche is repopulated with new HSCs. However, most patients lack compatible donors for HSCT, but cells derived from cord blood tend to be a more universal match (29, 30). Using HSCs derived from cord blood for transplant can help repopulate the BM, reduce the risk of rejection, and decrease disease relapse (11). However, HSCT therapy using cord blood cells is restricted by limited availability of donors (15), as well as by the absolute numbers of stem cells present in each collected sample. Polymer-based expansion methods provide innovative means to overcome these limitations by expanding stem cell populations from cord blood, or other hematopoietic stem cell sources, to potentially increase HSCT access.

**Figure 1:**
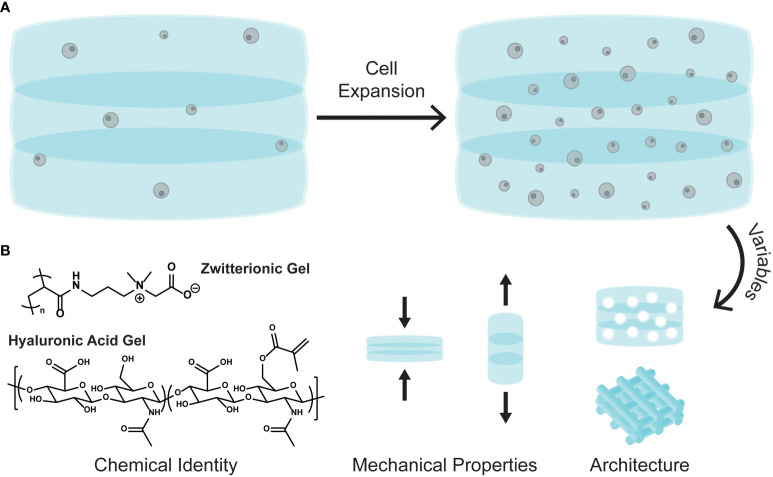
**(A)** Cartoon schematic of cell expansion in a 3D polymer matrix. **(B)** The tunable variables of 3D matrices: chemical identity, mechanical properties, and bulk architecture.

3D polymer-based cell matrices have been gaining in popularity for stem cell culture and expansion (31–35). Increasing attention focuses on the numerous tunable variables that can be incorporated in a polymer matrix, such as chemical identities, mechanical properties, and bulk matrix architecture (**Figure 1**). The diverse structures allow for discrete environmental manipulation and can tolerate cytokine and growth factor loading, which in turn affects cell growth, function, and differentiation (34).

A recent study explored the expansion of CD34+ cells isolated from cord blood in a 3D polymer matrix (15). Here the authors use a zwitterionic hydrogel composed of poly(carboxybetaine) and crosslinked using click chemistry and degradable crosslinkers. Their designed zwitterionic gel (ZG), which incorporates both negative and positive charges in a single monomer unit, significantly outperformed gels made from poly(ethylene glycol) (PEG), a common non-charged polymer used for many biological applications. In additional grafting studies, cells cultured in the ZGs showed similar engraftment levels than the non-cultured controls, but with 100-fold fewer cells. The authors ultimately show clinically meaningful expansion both of CD34+ cells isolated from cord blood and bone marrow derived HSPCs in their 3D zwitterionic hydrogel.

Modulating matrix mechanical properties, such as stiffness and elasticity, also plays a significant role in stem cell differentiation and expansion (36). One study demonstrated how polyacrylamide gels with differing Young’s moduli (E, stiffness) affected mesenchymal stem cell differentiation (MSC). For example, softer gels (E = 0.1-1 kPa) promoted neuronal type expression, while stiffer gels (E = 25-40 kPa) promoted osteogenic differentiation (37). Gels can also be synthesized to have dynamic moduli, where the stiffness changes when exposed to certain stimuli, such as light, pH, or temperature. These gels allow for cellular adaptation, being able to differentiate into various cell types by simply changing the matrix modulus (38).

Tuning the bulk matrix architecture offers another variable for control. The woodpile structure (**Figure 1B**, architecture) is commonly employed in 3D matrices as it can be easily manipulated to include diverse pore sizes and surface areas, both of which are important for cell growth and migration (25). A recent study investigated how gap sizes in a woodpile 3D matrix significantly affected bone marrow derived MSC (BM-MSC) migration (39). Matrices with larger gap sizes (100 μm) promoted the highest BM-MSC migration and increased the number of viable cells. Additionally, 3D cell matrices with submillimeter pore sizes were found to enhance CD34+ cell proliferation more than the nonporous matrices (33).

### 2.2 Therapeutic agent delivery

**Figure 2:**
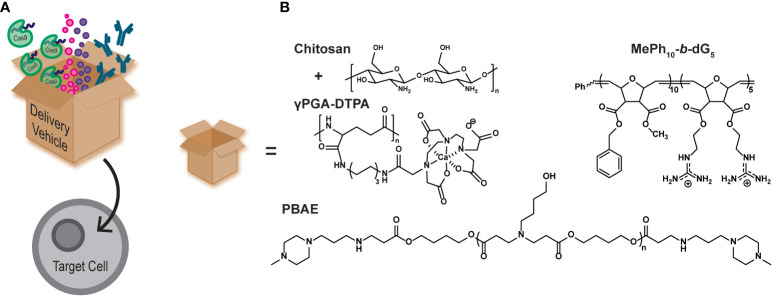
**(A)** Cartoon schematic of polymer delivery vehicles (represented by the box) being able to intracellularly deliver therapeutic cargo, such as gene editing tools (CRISPR/Cas9), small molecule drugs, and antibodies. **(B)** Examples of polymer delivery vehicles discussed in this mini review.

Polymers can also be used as intracellular delivery vehicles for drugs and biomacromolecules to aid in cellular manipulation (**Figure 2A**) (28). They are typically employed to help therapeutic cargo traverse the cell membrane, either by direct conjugation of the vehicle to the cargo, or by non-covalent complexation (40–42). Polymer-based delivery vehicles can also be used to protect the cargo from premature consumption or degradation. This aids in cargo pharmacokinetics, typically by increasing the half-life of the cargo or by increasing the toxicity threshold (27, 43).

Aplastic anemia (AA) is a form of bone marrow failure that is typically inherited, but can also be caused by drugs, such as hepatitis-C (HCV) treatments, and by diseases, such as the human immunodeficiency virus (HIV) virus. It is characterized by the bone marrow being incapable of producing enough blood cells for normal bodily functions. Filgrastim is a granulocyte colony-stimulating factor (G-CSF) used for hematopoietic cell growth and is known to treat neutropenia, aplastic anemia, and aids in myelosuppression after bone marrow transplantation (44–46). However, G-CSF is administered by daily injections, which is painful for patients and increases the risk of infection at the injection site (44). One study has shown the oral delivery of G-CSF mediated by a diethylene triamine penta acetic acid conjugated chitosan and poly(γ-glutamic acid) (γPGA-DTPA) nanoparticle (**Figure 2B**, chitosan+γPGA-DTPA) (44, 47). They observed increased availability of G-CSF when encapsulated in the nanoparticle and delayed the maximum concentration release by 6 h, indicative of a sustained release rather than a burst release. Additionally, neutropenia rat models were treated with a one-time oral dose of the nanoparticle encapsulated G-CSF (NP-G-CSF) or free-form G-CSF administered by daily injections. Both treatments increased the absolute neutrophil count in the rats, but the NP-G-CSF was only administered once, demonstrating the simplicity and effectiveness of the nanoparticle platform to deliver G-CSF.

Alternative ISTs must be utilized to ensure patients have safe and effective immune suppression without the occurrence of drug resistance and dose related toxicity (12). One strategy to achieve immunosuppression is to enhance regulatory T cell (Treg) function. Tregs are T cells that suppress effector cell growth and division, thereby limiting the immune response (48). FOXP3 is the hallmark protein for Treg cell function, whereby elevated levels correlate with enhanced suppressive function by Tregs (48–50).

It has been shown that protein kinase C theta (PKCθ) enhances effector T cell function, while limiting Treg differentiation. Many studies have sought to inhibit PKCθ function by using small molecule inhibitors or by using gene editing technology, such as siRNA. Inhibiting PKCθ yielded more suppressive Tregs and even restored impaired Treg function (51). Alternatively, polymer materials can be used as delivery vehicles for inhibitory biomacromolecules, such as antibodies, providing a simple pathway to achieve similar results. A recent study highlights a designed polymer vehicle to intracellularly deliver an antibody against PKCθ into naïve CD4+ T cells and inhibit its function (16). This polymer mimics the structure of the designed protein transduction domain, Pep-1 (Chariot^Ⓡ^), by using a block copolymer architecture with both hydrophobic and cationic blocks (**Figure 2B**, MePh_10_-*b*-dG_5_) (52). This polymer has also been shown to significantly outperform Pep-1 (53), an analog of the naturally occurring cell penetrating peptide HIV1-TAT (54), and the commercial delivery agent AbDeliverIN™ (55). The MePh_10_-*b*-dG_5_ delivery vehicle successfully delivered an anti-PKCθ antibody, and yielded Tregs with elevated FOXP3 expression and suppressive function (16). The exact mechanism by which these polymers carry cargo across the cell membrane to enhance the therapeutic agent delivery continues to be an area of active investigation.

Gene therapy is another avenue for protein manipulation, typically achieved by viral deliveries, such as lentivirus and extracellular vesicles, or nonviral deliveries, such as microinjection, electroporation, and polymeric vehicles (56). Viral delivery vectors face significant limitations as they pose mutagenetic risks and suffer from poor production yields (57). Microinjection and electroporation are popular nonviral delivery methods for genetic materials, but are harsh on cells, difficult to apply to large cell populations, and not feasible for *in vivo* work, making these techniques inadequate for clinical applications (56, 58, 59). Polymers such as poly(ethyleneimine), poly(amidoamine), and poly(amino acids), are promising as nonviral alternatives and have been successful at intracellularly delivering DNA, mRNA, and other gene editing technology (26, 60–62).

The CRISPR/Cas9 system has been widely adopted as an efficient and effective gene editing technology (63, 64). Polymeric delivery vehicles can be tailor-made for the desired cargo making them ideal vehicles for intracellular CRISPR/Cas9 delivery. Recently, poly(β-amino esters) (PBAEs) were synthesized to encapsulate the CRISPR/Cas9 system and transfect human CD34+ and CD14+ cells (**Figure 2B**, PBAE) (17). The transfection efficiency of the CRISPR/Cas9/PBAE nanoparticles (NPs) were >90% and maintained >86% cell viability. The NPs achieved 85% gene editing efficiency, which was measured by CD33 knockout. The edited cells were then injected into mice and the human cell engraftment was measured. Overall, there was no difference in human cell engraftment from the untreated control, both in the peripheral blood and the bone marrow of the mice (17).

## 3 Concluding remarks and perspective

Polymer materials are becoming increasingly popular to combat BMF because of their diverse chemical libraries, unique architecture, and relatively easy synthesis techniques. Here we have briefly highlighted some recent advances in these materials, serving as 3D cell matrices or as delivery vehicles for therapeutic cargo. Though these advancements are promising, there are other factors that need to be considered. The BM niche is a complex microenvironment that is difficult to accurately mimic synthetically. Incorporating cytokines, growth factors, and other BM niche components complicates the design for recreating a high-fidelity matrix, as well as for effective cell expansion. Improvements are also desperately needed to make these additional components compatible with accessible matrix synthesis techniques, such as 3D bioprinting.

Targeting intracellular pathways that contribute to enhanced Treg suppressive function have been shown to be promising therapeutic strategies. Increased expression of specific proteins like FOXP3, PRMT5, PD-1, and CTLA4 characterize highly suppressive Tregs, and targeting these proteins or their pathways have the potential to optimize outcomes in adoptive Treg therapy (65). These and other proteins and signaling pathways are accessible to polymers with cell penetrating properties that can deliver gene editing tools (i.e. CRISPR/Cas9 or siRNA) or signaling disrupting agents (i.e. inhibitory antibodies). Additionally, the BM microenvironment has several unique enzymes, such as serine protease 57, elastase, neutrophil expressed bactericidal permeability increasing protein, defensin alpha 3, ribonuclease A family member 3, and surface receptors such as olfactory receptor family 10 subfamily Z member 1 (Atlas database). If polymer materials can be modified to specifically target these unique elements, then therapeutic drugs will be ensured to solely reach the BM and thus limits off-target toxicity, which is common in most small molecule therapeutics. Overall, expanding the use of polymer materials promises to improve BMF therapies by enhancing stem cell expansion and as delivery vehicles for therapeutic agents.

## Author contributions

KK: Writing – original draft, Writing – review & editing. NJ: Writing – original draft, Writing – review & editing. IT: Writing – original draft, Writing – review & editing. GT: Funding acquisition, Writing – review & editing. LM: Writing – review & editing.

## References

[1] Bone Marrow Failure - StatPearls - NCBI Bookshelf . Available online at: https://www.ncbi.nlm.nih.gov/books/NBK459249/?report=reader (Accessed January 3, 2024).

[2] Laughlin MJ Barker J Bambach B Koc ON Rizzieri DA Wagner JE. Hematopoietic engraftment and survival in adult recipients of umbilical-cord blood from unrelated donors. N Engl J Med. (2001) 344:1815–22. doi: 10.1056/NEJM200106143442402 11407342

[3] Bacigalupo A Socie’ G Lanino E Prete A Locatelli F Locasciulli A. Fludarabine, cyclophosphamide, antithymocyte globulin, with or without low dose total body irradiation, for alternative donor transplants, in acquired severe aplastic anemia: a retrospective study from the EBMT-SAA working party. Haematologica. (2010) 95:976–82. doi: 10.3324/HAEMATOL.2009.018267 PMC2878797 20494932

[4] Anderlini P Wu J Gersten I Ewell M Tolar J Antin JH. Cyclophosphamide conditioning in patients with severe aplastic anemia given unrelated marrow transplantation: a phase 1–2 dose de-escalation study. Lancet Hematol. (2015) 2:e367. doi: 10.1016/S2352-3026(15)00147-7 PMC4861234 26685770

[5] De Latour RP. Transplantation for bone marrow failure: current issues. Hematol Am Soc Hematol Educ Progr. (2016) 2016:90–8. doi: 10.1182/ASHEDUCATION-2016.1.90 PMC6142500 27913467

[6] Tang Y Desierto MJ Chen J Young NS. The role of the Th1 transcription factor T-bet in a mouse model of immune-mediated bone-marrow failure. Blood. (2010) 115:541. doi: 10.1182/BLOOD-2009-03-211383 19903901 PMC2810980

[7] Roderick JELM Gonzalez-Perez G Kuksin CA Dongre A Roberts ER Srinivasan J. Therapeutic targeting of NOTCH signaling ameliorates immune-mediated bone marrow failure of aplastic anemia. J Exp Med. (2013) 210:1311. doi: 10.1084/JEM.20112615 23733784 PMC3698520

[8] Kurtzberg J Laughlin M Graham ML Smith C Olson JF Halperin EC. Placental blood as a source of hematopoietic stem cells for transplantation into unrelated recipients. N Engl J Med. (1996) 335:157–66. doi: 10.1056/NEJM199607183350303 8657213

[9] Gupta V Eapen M Brazauskas R Carreras J Aljurf M Gale RP. Impact of age on outcomes after bone marrow transplantation for acquired aplastic anemia using HLA-matched sibling donors. Haematologica. (2010) 95:2119–25. doi: 10.3324/HAEMATOL.2010.026682 PMC2995571 20851870

[10] de Lima M McNiece I Robinson SN Munsell M Eapen M Horowitz M. Cord-blood engraftment with ex vivo mesenchymal-cell coculture. N Engl J Med. (2012) 367:2305–15. doi: 10.1056/NEJMOA1207285 PMC3805360 23234514

[11] Milano F Gooley T Wood B Woolfrey A Flowers ME Doney K. Cord-blood transplantation in patients with minimal residual disease. N Engl J Med. (2016) 375:944–53. doi: 10.1056/NEJMoa1602074 PMC5513721 27602666

[12] Shah S Jain P Shah K Patel K Parikh S Patel A. Immunosuppressive therapy for aplastic anemia: a single-center experience from western India. Ann Hematol. (2019) 98:41. doi: 10.1007/S00277-018-3487-2 30173288 PMC6334724

[13] Patel A Patel A Parikh S Shah S Panchal H Anand A. Acquired severe aplastic anemia treated with antithymocyte globulin and cyclosporine: An experience of regional cancer center, Western India. J Appl Hematol. (2015) 6:53. doi: 10.4103/1658-5127.160198

[14] Wilkinson AC Igarashi KJ Nakauchi H. Hematopoietic stem cell self-renewal in *vivo* and ex vivo. Nat Rev Genet. (2020) 21:541–54. doi: 10.1038/s41576-020-0241-0 PMC7894993 32467607

[15] Bai T Li J Sinclair A Imren S Merriam F Sun F. Expansion of primitive human hematopoietic stem cells by culture in a zwitterionic hydrogel. Nat Med. (2019) 25:1566–75. doi: 10.1038/s41591-019-0601-5 31591594

[16] Ozay EI Shanthalingam S Sherman HL Torres JA Osborne BA Tew GN. Cell-penetrating anti-protein kinase C theta antibodies act intracellularly to generate stable, highly suppressive regulatory T cells. Mol Ther. (2020) 28:1987–2006. doi: 10.1016/J.YMTHE.2020.05.020 32492367 PMC7474270

[17] El-Kharrag R Berckmueller KE Madhu R Cui M Campoy G Mack HM. Efficient polymer nanoparticle-mediated delivery of gene editing reagents into human hematopoietic stem and progenitor cells. Mol Ther. (2022) 30:2186–98. doi: 10.1016/j.ymthe.2022.02.026 PMC9171380 35240320

[18] Sgolastra F Kuksin CA Gonzalez-Perez G Minter LM Tew GN. Enhanced TAT-cre protein transduction for efficient gene recombination in T cells. ACS Appl Bio Mater. (2018) 1:444–51. doi: 10.1021/acsabm.8b00153 35016365

[19] Spychalska K Zajac D Baluta S Halicka K Cabaj J. Functional polymers structures for (Bio)Sensing application—A review. Polymers (Basel). (2020) 12. doi: 10.3390/POLYM12051154 PMC7285029 32443618

[20] Zhu Y Liu W Ngai T. Polymer coatings on magnesium-based implants for orthopedic applications. J Polym Sci. (2022) 60:32–51. doi: 10.1002/POL.20210578

[21] Li H Sun J Zhu H Wu H Zhang H Gu Z. Recent advances in development of dendritic polymer-based nanomedicines for cancer diagnosis. Wiley Interdiscip Rev Nanomedicine Nanobiotechnology. (2021) 13:e1670. doi: 10.1002/WNAN.1670 32949116

[22] Tezgel AÖ Telfer JC Tew GN. *De novo* designed protein transduction domain mimics from simple synthetic polymers. Biomacromolecules. (2011) 12:3078–83. doi: 10.1021/bm200694u 21714570

[23] Feng W Huang Z Kang X Zhao D Li H Li G. Self-assembled nanosized vehicles from amino acid-based amphiphilic polymers with pendent carboxyl groups for efficient drug delivery. Biomacromolecules. (2021) 22:4871–82. doi: 10.1021/acs.biomac.1c01164 34636237

[24] Wang MQ Zou H Bin LW Liu N Wu ZQ. Bottlebrush polymers based on RAFT and the “c1” Polymerization method: controlled synthesis and application in anticancer drug delivery. ACS Macro Lett. (2022) 11:179–85. doi: 10.1021/acsmacrolett.1c00706 35574766

[25] Raic A Rödling L Kalbacher H Lee-Thedieck C. Biomimetic macroporous PEG hydrogels as 3D scaffolds for the multiplication of human hematopoietic stem and progenitor cells. Biomaterials. (2014) 35:929–40. doi: 10.1016/J.BIOMATERIALS.2013.10.038 24176196

[26] Huang P Deng H Zhou Y Chen X. The roles of polymers in mRNA delivery. Matter. (2022) 5:1670–99. doi: 10.1016/J.MATT.2022.03.006

[27] Liechty WB Kryscio DR Slaughter BV Peppas NA. Polymers for drug delivery systems. Annu Rev Chem Biomol Eng. (2010) 1:149. doi: 10.1146/ANNUREV-CHEMBIOENG-073009-100847 22432577 PMC3438887

[28] Lv J Fan Q Wang H Cheng Y. Polymers for cytosolic protein delivery. Biomaterials. (2019) 218:119358. doi: 10.1016/j.biomaterials.2019.119358 31349095

[29] Calado RT Clé DV. Treatment of inherited bone marrow failure syndromes beyond transplantation. Hematol Am Soc Hematol Educ Progr. (2017) 2017:96. doi: 10.1182/ASHEDUCATION-2017.1.96 PMC6142589 29222242

[30] Ballen KK Gluckman E Broxmeyer HE. Umbilical cord blood transplantation: the first 25 years and beyond. Blood. (2013) 122:491. doi: 10.1182/BLOOD-2013-02-453175 23673863 PMC3952633

[31] Xu Y Chen C Hellwarth PB Bao X. Biomaterials for stem cell engineering and biomanufacturing. Bioact Mater. (2019) 4:366–79. doi: 10.1016/J.BIOACTMAT.2019.11.002 PMC6909203 31872161

[32] Eskandari F Allahverdi A Nasiri H Azad M Kalantari N Soleimani M. Nanofiber expansion of umbilical cord blood hematopoietic stem cells. Iran J Pediatr Hematol Oncol. (2015) 5:170. PMC4779151 26985349

[33] Liu B Jin M Wang DA. *In vitro* expansion of hematopoietic stem cells in a porous hydrogel-based 3D culture system. Acta Biomater. (2023) 161:67–79. doi: 10.1016/J.ACTBIO.2023.01.057 36754271

[34] Liu B Tao C Wu Z Yao H Wang DA. Engineering strategies to achieve efficient in *vitro* expansion of hematopoietic stem cells: development and improvement. J Mater Chem B. (2022) 10:1734–53. doi: 10.1039/D1TB02706A 35191442

[35] Lee Y Arai Y Ahn J Kim D Oh S Kang D. Three-dimensional microenvironmental priming of human mesenchymal stem cells in hydrogels facilitates efficient and rapid retroviral gene transduction via accelerated cell cycle synchronization. NPG Asia Mater. (2019) 11:1–11. doi: 10.1038/s41427-019-0127-9

[36] Han S-B Kim J-K Lee G Kim D-H Han S-B Kim J-K. Mechanical properties of materials for stem cell differentiation. Adv Biosyst. (2020) 4:2000247. doi: 10.1002/ADBI.202000247 33035411

[37] Wen JH Vincent LG Fuhrmann A Choi YS Hribar KC Taylor-Weiner H. Interplay of matrix stiffness and protein tethering in stem cell differentiation. Nat Mater. (2014) 13:979–87. doi: 10.1038/NMAT4051 PMC4172528 25108614

[38] Lin CH Su JJM Lee SY Lin YM. Stiffness modification of photopolymerizable gelatin-methacrylate hydrogels influences endothelial differentiation of human mesenchymal stem cells. J Tissue Eng Regener Med. (2018) 12:2099–111. doi: 10.1002/TERM.2745 30058281

[39] Costa BNL Adão RMR Maibohm C Accardo A Cardoso VF Nieder JB. Cellular interaction of bone marrow mesenchymal stem cells with polymer and hydrogel 3D microscaffold templates. ACS Appl Mater Interfaces. (2022) 14:13013–24. doi: 10.1021/ACSAMI.1C23442 PMC8949723 35282678

[40] Dorogin J Townsend JM Hettiaratchi MH. Biomaterials Science REVIEW Biomaterials for protein delivery for complex tissue healing responses. Cite this Biomater Sci. (2021) 9:2339. doi: 10.1039/d0bm01804j 33432960

[41] Kalafatovic D Giralt E. Cell-penetrating peptides: design strategies beyond primary structure and amphipathicity. Mol. (2017) 22:1929. doi: 10.3390/MOLECULES22111929 PMC6150340 29117144

[42] Ramakrishna S Kwaku Dad AB Beloor J Gopalappa R Lee SK Kim H. Gene disruption by cell-penetrating peptide-mediated delivery of Cas9 protein and guide RNA. Genome Res. (2014) 24:1020–7. doi: 10.1101/GR.171264.113 PMC4032848 24696462

[43] Varela-Moreira A van Straten D van Leur HF Ruiter RWJ Deshantri AK Hennink WE. Polymeric micelles loaded with carfilzomib increase tolerability in a humanized bone marrow-like scaffold mouse model. Int J Pharm X. (2020) 2:100049. doi: 10.1016/J.IJPX.2020.100049 32490374 PMC7262453

[44] Su F-Y Chuang E-Y Lin P-Y Chou Y-C Chen C-T Mi F-L. Treatment of chemotherapy-induced neutropenia in a rat model by using multiple daily doses of oral administration of G-CSF-containing nanoparticles. Biomaterials. (2014) 35:3641–9. doi: 10.1016/j.biomaterials.2014.01.020 24477192

[45] Welte K Gabrilove J Bronchud MH Platzer E Morstyn G. Filgrastim (r-metHuG-CSF): the first 10 years. Blood. (1996) 88:1907–29. doi: 10.1182/BLOOD.V88.6.1907.BLOODJOURNAL8861907 8822908

[46] Wang Y Zhao C Ma P Jiang D. Outcome and cost-effectiveness analysis of long-acting G-CSF as primary prophylaxis of neutropenia induced by chemotherapy in breast cancer patients, from a retrospective study. Cancer Control. (2023) 30. doi: 10.1177/10732748221140289/ASSET/IMAGES/LARGE/10.1177_10732748221140289-FIG5.JPEG PMC9827532 36598048

[47] Su FY Lin KJ Sonaje K Wey SP Yen TC Ho YC. Protease inhibition and absorption enhancement by functional nanoparticles for effective oral insulin delivery. Biomaterials. (2012) 33:2801–11. doi: 10.1016/J.BIOMATERIALS.2011.12.038 22243802

[48] Plitas G Rudensky AY. Regulatory T cells: differentiation and function. Cancer Immunol Res. (2016) 4:721. doi: 10.1158/2326-6066.CIR-16-0193 27590281 PMC5026325

[49] Fontenot JD Gavin MA Rudensky AY. Foxp3 programs the development and function of CD4+CD25+ regulatory T cells. Nat Immunol. (2003) 4:986–92. doi: 10.1038/NI904 28115587

[50] Rudensky AY. Regulatory T cells and foxp3. Immunol Rev. (2011) 241:260–8. doi: 10.1111/J.1600-065X.2011.01018.X PMC3077798 21488902

[51] Boschelli D. Small molecule inhibitors of PKCθ; as potential antiinflammatory therapeutics. Curr Top Med Chem. (2009) 9:640–54. doi: 10.2174/156802609789007372 19689371

[52] Ramsey JD Flynn NH. Cell-penetrating peptides transport therapeutics into cells. Pharmacol Ther. (2015) 154:78–86. doi: 10.1016/J.PHARMTHERA.2015.07.003 26210404

[53] Backlund CM Hango CR Minter LM Tew GN. Protein and antibody delivery into difficult-to-transfect cells by polymeric peptide mimics. ACS Appl Bio Mater. (2020) 3:180–5. doi: 10.1021/acsabm.9b00876 35019434

[54] Backlund CM Takeuchi T Futaki S Tew GN. Relating structure and internalization for ROMP-based protein mimics. Biochim Biophys Acta. (2016) 1858:1443. doi: 10.1016/J.BBAMEM.2016.03.024 27039278 PMC4964965

[55] Ozay EI Gonzalez-Perez G Torres JA Vijayaraghavan J Lawlor R Sherman HL. Intracellular delivery of anti-pPKCθ (Thr538) via protein transduction domain mimics for immunomodulation. Mol Ther. (2016) 24:2118–30. doi: 10.1038/mt.2016.177 PMC5167783 27633441

[56] Yip BH. Recent advances in CRISPR/cas9 delivery strategies. Biomolecules. (2020) 10. doi: 10.3390/BIOM10060839 PMC7356196 32486234

[57] Eliyahu H Barenholz Y Domb AJ. Polymers for DNA delivery. Molecules. (2005) 10:34–64. doi: 10.3390/10010034 18007276 PMC6147627

[58] Koch KC Tew GN. Functional antibody delivery: Advances in cellular manipulation. Adv Drug Delivery Rev. (2023) 192:114586. doi: 10.1016/J.ADDR.2022.114586 36280179

[59] Zhang Y Yu LC. Microinjection as a tool of mechanical delivery. Curr Opin Biotechnol. (2008) 19:506–10. doi: 10.1016/J.COPBIO.2008.07.005 18725294

[60] Thomas TJ Tajmir-Riahi HA Pillai CKS. Biodegradable polymers for gene delivery. Molecules. (2019) 24. doi: 10.3390/MOLECULES24203744 PMC6832905 31627389

[61] Chen W Ma Y Liu X Zhu D. Polyester materials for mRNA delivery. Open Explor 2019 32. (2022) 3:117–27. doi: 10.37349/ETAT.2022.00075 PMC9400784 36046844

[62] Heo MB Cho MY Lim YT. Polymer nanoparticles for enhanced immune response: Combined delivery of tumor antigen and small interference RNA for immunosuppressive gene to dendritic cells. Acta Biomater. (2014) 10:2169–76. doi: 10.1016/j.actbio.2013.12.050 24394635

[63] Gostimskaya I. CRISPR–cas9: A history of its discovery and ethical considerations of its use in genome editing. Biochem (Mosc). (2022) 87:777. doi: 10.1134/S0006297922080090 PMC9377665 36171658

[64] Ishino Y Krupovic M Forterre P. History of CRISPR-cas from encounter with a mysterious repeated sequence to genome editing technology. J Bacteriol. (2018) 200. doi: 10.1128/JB.00580-17 PMC5847661 29358495

[65] Jadon N Shanthalingam S Tew GN Minter LM. PRMT5 regulates epigenetic changes in suppressive Th1-like iTregs in response to IL-12 treatment. (2024) 14. doi: 10.3389/fimmu.2023.1292049 PMC10800960 38259494

